# Time-Restricted Feeding in Commercial Layer Chickens Improves Egg Quality in Old Age and Points to Lack of Adipostat Activity in Chickens

**DOI:** 10.3389/fphys.2021.651738

**Published:** 2021-06-21

**Authors:** Ganesan Saibaba, Mark Ruzal, Dima Shinder, Sara Yosefi, Shelly Druyan, Hagit Arazi, Oren Froy, Dror Sagi, Miriam Friedman-Einat

**Affiliations:** ^1^Institute of Animal Science, Agricultural Research Organization (ARO), Volcani Center, Rishon Lezion, Israel; ^2^Extension Service, Poultry Division, Ministry of Agriculture and Rural Development, Beit Dagan, Israel; ^3^Robert H. Smith Faculty of Agriculture, Food and Environment, Institute of Biochemistry, Food Science and Nutrition, The Hebrew University of Jerusalem, Rehovot, Israel

**Keywords:** time-restricted feeding, chicken, egg production, egg quality, appetite control

## Abstract

In mammals, time-restricted feeding (TRF) with no caloric restriction provides health benefits and extends longevity, usually with a minor (∼3%) or no reduction in total food consumption. In the current study, a TRF regimen of 6 h free access to food (08:00–14:00 h) was applied to Leghorn chickens from 25 to 86 weeks of age; control birds ate freely during the light hours (06:00–20:00 h). Unexpectedly, the TRF-treated birds consumed, on average, 11.7% less food than the controls. This was manifested by an average reduction of 9.6% in body weight, 2.6-fold in visceral fat accumulation, and 6.5% in egg weight. Hen-housed egg production was reduced by 3.6% in the TRF group compared with the control, along the first 40 weeks of the follow-up (*P* < 0.05), and changed into a tendency of 0.7% higher egg production thereafter. Several parameters of egg quality showed significant improvement (*P* < 0.05) in the TRF group compared with the controls. A comparison of diurnal patterns of feed consumption revealed a higher rate of hourly consumption in the TRF group and increased consumption before dark in the control group. In conclusion, the reduced feed intake in response to the TRF treatment and loss in visceral fat accumulation supports the lack of a strong adipostat activity in chickens and different appetite regulation mechanisms compared with mammals. Therefore, future TRF studies in chickens should be adjusted by extending the *ad libitum* time window. The lower feed intake by the TRF-treated chickens compared with the *ad libitum*-fed controls seems to reduce the efficiency of egg production. Nevertheless, the improved egg quality and persistence of egg lay at the older age suggest that similarly to mammals, the TRF treatment delayed at least some of the negative impacts associated with advanced age.

## Introduction

Time-restricted feeding (TRF), defined as food consumed for ≤10 h per day, extends the time spent fasting and improves markers of metabolic health in both animal models (Froy and Miskin, 2010; Hatori et al., 2012) and humans (Sutton et al., 2018; Jones et al., 2020). In mammals, the improved health, metabolism, reproductive efficiency, brain signaling, and longevity were reported by many studies, with caloric intake similar to the *ad libitum* control group (Froy and Miskin, 2010; Hatori et al., 2012; Manoogian and Panda, 2017; Balasubramanian et al., 2020; Currenti et al., 2020; Hua et al., 2020). A global “experiment” in humans is Ramadan, a Muslim observance that consists of fasting from sunrise to sunset for a month: this practice has limited or no effect on body weight (BW) or body composition while improving blood lipid profile, insulin sensitivity, and other metabolic parameters; these beneficial physiological effects usually last beyond the Ramadan period (Osman et al., 2020). At the molecular level, the effects of TRF are tightly correlated with attenuation of the age-related decline in the phase and amplitude of circadian genes (also called clock genes) (Froy, 2011). We have shown that long-term day-time TRF in mice can increase the amplitude of clock-gene expression, increase the expression of catabolic factors, and reduce the levels of disease markers leading to better health (Sherman et al., 2011). Moreover, a recent study employing mouse mutants showed that TRF protects against the metabolic defects stemming from clock-gene mutations (Chaix et al., 2019). Taken together, evidence indicates complex interactive regulation between the control mechanisms implicated in the TRF response and circadian rhythms.

Biological clock function, which controls daily changes in sleeping and waking, visual function, song, migratory patterns and orientation, and seasonal patterns of reproduction, has been investigated in birds for over a century (Schäfer, 1907). The central pacemakers in the avian pineal gland, retinae, and suprachiasmatic nucleus (SCN) of the hypothalamus interact dynamically to maintain stable phase relationships and influence downstream rhythms through entrainment of peripheral oscillators in the brain controlling behavior and peripheral tissues [(Cassone, 2014) and references therein]. In mammals, the SCN is the predominant central pacemaker of the circadian clock (Bechtold and Loudon, 2013).

Birds present a good model for the role played by biological clocks in humans because unlike most rodent models, they are diurnal, they exhibit cognitively complex social interactions, and their circadian clock is more sensitive to the hormone melatonin than those of nocturnal rodents (Cassone, 2014). From an evolutionary perspective, birds represent an important clade, bridging the gap between reptiles and mammals. A practical advantage of using laying hens for such studies is that the rates of egg laying and egg quality decline with age and are easy to follow. The domestication of chickens ∼4,500 years ago and extensive selective breeding primarily in the last century have greatly enhanced egg production in the commercial layer. Whereas the wild-type chicken (represented by red and gray junglefowl) usually lays a single clutch of 5–9 eggs a year (Qanbari et al., 2019), today’s commercial layer chicken lays more than 320 eggs in the first year of lay. This extremely high laying efficiency gradually declines with age, reaching a level that is below commercial efficiency (70% production or less), within the second year of age. Therefore, the study of long-term TRF in layer hens provides a new model system with unique follow-up tools, which are of high interest to both academic and agricultural research.

## Materials and Methods

### Animals

Female Leghorn chicks (white shell egg Lohmann-type layers) were purchased from a commercial poultry farm (Hasolelim, Israel) at 1 day of age. The chicks were grown on floor pens containing wood shavings and a chick density of 200 chicks in a 4 × 5-meter room. Gas warming started at 34°C on the arrival day and gradually decreased to 24°C during the first 28 days. Light management was according to the Lohmann guide^1^. Feed formulated according to the (National Research Council (NRC), 1994); pre-starter 0–4 weeks of age (WOA), starter 4–8 WOA, pullets 8–15 WOA] recommendations was purchased from Brown and Sons (Hod Hasharon, Israel). Water and feeding were provided for *ad libitum* consumption.

### Experimental Design

At the age of 15 weeks, the birds were divided into two groups with similar average BW and BW distribution. Each group was divided into 10 subgroups of 8 birds (total of 160 birds) and transferred to individual cages, sized 40 × 40 × 45 (height) cm, each with access to two nipple drinkers in an open house setting. The 10 subgroups were distributed in a randomized design for equal exposure to microenvironments in the different parts of the shed. At 19 WOA, artificial lighting was gradually applied to allow 14 h:10 h light:dark (light from 06:00 to 20:00 h) by artificial electric lighting in addition to the natural light–dark cycle in weekly increments of half hour in the morning and in the evening. The physiological parameters of entry into egg lay, before the beginning of the TRF treatment, are summarized in Table 1. Average BW, weight of the first laid egg, and age of the bird at first egg lay were similar for the two groups (Table 2). The TRF treatment was gradually applied at 25 WOA. Feeders were covered to prevent eating in daily steps of 1–6 h of free feeding (08:00–14:00 h), and this regimen was maintained until the end of the experiment (86 WOA). Feeding formula (layer formula) was constant from 15 to 86 WOA; its content is detailed in Figure 2.

**TABLE 1:** Measures of entry to lay of the two groups before TRF treatment.

	**Mean control**	**Mean TRF**	***P*-value**
BW at first egg (g)	1,501.1 ± 13.9	1,501.6 ± 10.6	0.91
First egg weight (g)	43.9 ± 0.5	45.0 ± 0.3	0.08
Age at first egg (days)	140.8 ± 0.7	142.2 ± 0.7	0.29

*BW, body weight.*

**TABLE 2:** Feeding formula of layer (15–61 weeks of age).

**Ingredients**	**Amount**
Protein %	17
Calcium %	4
Phosphor %	0.5
Fat %	4.5
Ash %	12
Fiber %	4
Salt %	0.35
Manganese (gr)	80
Linoleic acid %	1.7
Nutritional content (kcal/kg)	2,750

**FIGURE 1:**
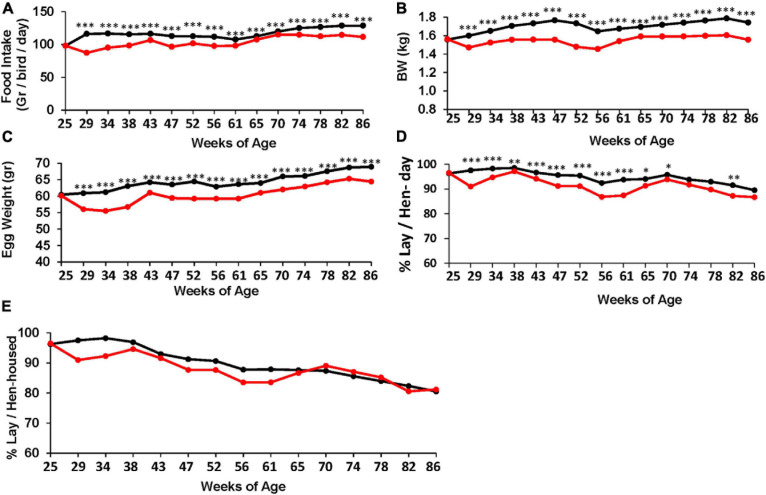
Effect of the TRF treatment on food intake and performance. **(A)** Feed intake *N* = 10. **(B)** Body weight. **(C)** Egg weight. **(D)** Laying efficiency, calculated per actual living bird (bird-day; 100% = 1 egg per bird per day). ^∗^, ^∗∗^, and ^∗∗∗^ represent *P*-values < 0.05, 0.01, and 0.001, respectively, obtained from *T*-test. Annual statistics are presented in Table 3. **(E)** Laying efficiency similar to **(D)** but calculated per the original number of birds at the beginning of the experiment (hen-housed). Statistical analysis for **(E)** is shown in Table 4. *N* = 80 for each treatment **(B–E)**. Black and red lines represent the control and TRF groups, respectively.

**TABLE 3:** Summary of the responses to TRF treatment (annual averages).

	**Control**	**Standard error**	**TRF**	**Standard error**	***P-*value**	**Fold difference**	**% Change**
						
						**TRF vs. *ad libitum***	***Ad libitum* vs. TRF**
Food intake*	117.8	2.1	104.0	2.7	8.7E−05	1.133	11.75
Food conversion rate**	1.92	0.03	1.91	0.03	8.0E−01	1.004	0.42
Body weight (g)	1,702	0.02	1,538	0.02	6.6E−05	1.107	9.64
% Egg lay (bird-day)	95.14	0.71	91.32	0.98	2.0E−03	1.042	3.82
Egg weight	64.63	0.78	60.43	0.98	4.4E−04	1.070	6.50

*Average food intake/day/bird.

**Food intake/egg mass.

**TABLE 4:** Dynamic changes in comparative laying efficiency between the TRF and control chickens, calculated per hen housed.

	**Mean of % lay**		
**Weeks of age**	**25−57**	**58−86**	**25−86**
Control	92.3	83.9	89.3
TRF	88.7	84.6	87.3
Control–TRF	3.6	−0.7	2.1
*P*-value*	0.0001	0.58	0.005

*P-values were obtained from log-rank test.

**FIGURE 2:**
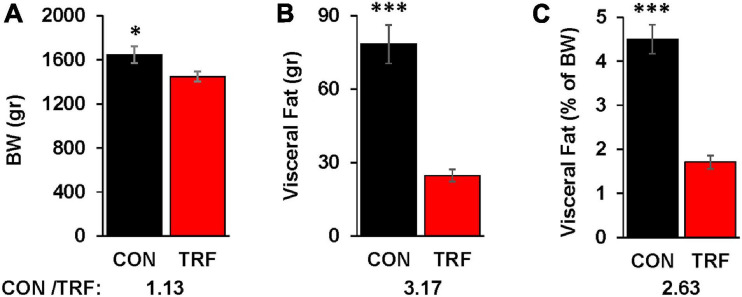
The effect of the TRF treatment on visceral fat accumulation. **(A)** Live body weight (BW) in the control (CON) and TRF hens of 14 randomly selected birds from each group at 86 WOA. **(B)** Visceral fat accumulation of the birds in **(A)**. **(C)** Percent visceral fat of live body weight [based on the data in **(A,B)**]. Ratio of values from control versus TRF-treated birds is shown at the bottom of each panel. Vertical lines represent scanning electron microscope. ^∗^, ^∗∗∗^*P* < 0.05 and 0.001, respectively.

### Egg Laying and Quality Follow-Up

Birds were weighed at the end of each month at 14:00 h (after lay time). Egg production and quality [except Haugh unit (HU) analysis, see below] were followed daily on an individual basis. This manual inspection included all egg laid during the experiment (a total of 29,694 and 28,910 eggs laid by the control and treated chickens, respectively). Food intake was measured for each group of eight birds at the end of each month, and daily food consumption per hen was calculated. Eggs were weighed on 2 consecutive days at the end of each month, for all eggs laid on those days. HU score of freshly laid eggs was measured at the last 2 days before beginning the time restriction feeding regimen (T0) and the last 2 days of the experiment and calculated as: HU = 100 × log(h − 1.7W^0.37^ + 7.57), where h = observed height of the albumen (mm) and W = egg weight (g). The height of the albumen was measured in open eggs using a digital apparatus (QCH; Technical Services and Supplies Ltd., Dunnington, England).

### Diurnal Pattern of Feed-Consumption Behavior

Along 1 day of the experiment (at 50 WOA), feeders were filled and weighed before exposing the control and TRF groups (starting at 06:00 and 08:00 h, respectively) to *ad libitum* feeding. Feeders were then weighed either every hour or every 15 min (as indicated) after the beginning and until the end of the daily eating (20:00 and 14:00 h for the control and TRF groups, respectively). For each group, seven feeders were employed at each feeding of four birds.

### Visceral Fat Accumulation

At the last day of the experiment, 14 randomly picked birds from each group were killed by neck dislocation. Intra-abdominal fat (visceral fat) was collected using forceps and weighted. Fat weight percentage of live BW was calculated.

### Statistical Analysis

Statistical analysis was performed by one-way ANOVA and Tukey–Kramer honestly significant difference test (*P* ≤ 0.05); mean ± standard error (SE) was reported. When indicated, analysis was performed using log-rank test, suited for measurements time-to-event data.

## Results

### Effect of TRF on Feed Intake and Egg Production

To assess the effect of the TRF regimen on egg-laying performance, Leghorn-type chickens were exposed to *ad libitum* feeding (control) or to limited access to feed of 6 h daily (TRF; 0800–14:00 h). The TRF hens consumed, on average, 75.2 and 81.4% compared with the *ad libitum*-fed hens in the first and second months of the experiment, respectively, and an average of 90.3% ± 1.0 SE thereafter (Figure 1A). The BW of the TRF chickens showed an average of 9.6% weight loss compared with the *ad libitum*-fed group but with no deeper weight loss in the first 2 months (Figure 1B), despite the lower feed intake. Egg weight showed significant reductions along the experiment and more so in the first 2 months (Figure 1C), corresponding to the higher reduction in food intake. Egg lay calculated per actual bird (hen-day; Figure 1D) showed that persistent laying was reduced by the treatment but was the least affected compared with BW and egg weight (Table 3 and Figure 1D). Interestingly, the effect on persistent laying was prominent until the age of 61 weeks. From 62 to 68 WOA, the reduced laying efficiency of the TRF birds was in some periods less prominent and in others not significant (Figure 1D). This tendency of improved egg lay at the older age of the TRF birds was even more pronounced when egg lay was calculated based on the original number of birds (hen-housed; Figure 1E). Due to a higher tendency of death in the control group than in the TRF group (7 versus 5, respectively), egg lay per hen housed was higher in the TRF group than in the control group at the older age, although by only 0.7% on average (Table 4). This switch from reduced to similar egg production of the TRF-treated birds compared with the controls suggests that the TRF treatment delayed the characteristic lowering of egg production associated with advanced chicken’s age.

Visceral fat was measured at the end of the experiment in 14 representative birds from each group (Figure 2). Whereas BW differed by 13%, visceral fat accumulation was reduced by 3.17-fold. Normalization of visceral fat to BW showed 2.6-fold less fat deposition in the TRF than in the control birds.

### Effect of TRF on Egg Quality

**FIGURE 3:**
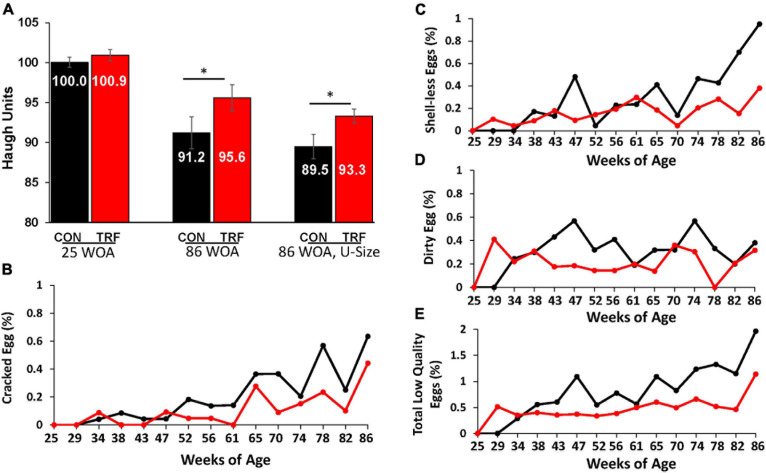
The effect of the TRF treatment on some parameters of egg quality. **(A)** Haugh unit (HU) scores were measured in eggs of the TRF and control groups the last 2 days before TRF treatment (24 WOA) and the last 2 days of the experiment (86 WOA). In both measurements, all eggs laid on the same day and 1 day prior (kept in a 20°C room) were included (*N* = 298 and 195, respectively). At the end of the experiment, the third comparison was of uniform size (U-size; *N* = 90 in each group). **(B–D)** Cracked, shell-less, and dirty eggs, respectively, as percentage of the total laid eggs in each group. **(E)** Percent of total low-quality eggs relative to total eggs laid (based on **B–D**). Black and red lines represent the control and TRF groups, respectively. Statistical analysis from log-rank test is shown in Table 5. *P > 0.05.

To estimate the possible effects of the TRF treatment on the hens’ physiology, we followed several aspects of egg quality, which significantly deteriorates with age (Molnar et al., 2016; Park and Sohn, 2018). One such classical measure is HU score, which indicates egg protein quality based on the height of the egg white. HU scores were similar between the TRF-treated and control groups before the TRF treatment; however, by the end of the experiment, significantly (*P* ≤ 0.05) higher HU scores were obtained for eggs laid by the TRF-treated birds (Figure 3A). Calculation of HU scores normalized the value to egg weight (see the Materials and Methods section). Nevertheless, to demonstrate that this result was not related to the lower eggs weight laid by the TRF-treated birds, we repeated the comparison using only similar-sized eggs of 66.7 g on average at a narrow range of egg weights (61.4–73.0 g; Figure 3). Significantly higher HU scores (*P* = 0.03) were also observed with these eggs for the TRF group. Altogether, this analysis showed that eggs laid by the TRF-treated birds had better protein quality at advanced age (Silversides and Scott, 2001).

**TABLE 5:** Effect of the TRF on the proportion of low-quality eggs.

**Egg type**	**Treatment**	Mean*	Standard error*	*P*-value*
Cracked	Control	0.32	0.06	0.0035
	TRF	0.16	0.04	
Shell-less	Control	0.40	0.09	0.0003
	TRF	0.21	0.03	
Dirty	Control	0.34	0.04	0.0043
	TRF	0.20	0.04	
Total low quality	Control	1.05	0.14	6.9e−08
	TRF	0.57	0.07	

*Values were calculated for 29–86 weeks of age. *P*-values were calculated using log-rank test.

As an additional characteristic of egg quality, we measured the proportion of cracked eggs, eggs lacking calcified eggshell (shell-less), and eggs with dirty shells (Figures 3B–E). The proportion of these types of low-quality eggs was lower in hens in the TRF group, from 29 WOA onward. It is likely that the effect on egg quality in the first few weeks of treatment resulted from slow adaptation to the feeding regimen, as indicated by the much lower feed intake. Statistical summary of the comparative egg quality is shown in Table 2. The parameters indicating low-quality eggs were significantly lower (*P* < 0.05) in the TRF group between 29 and 86 WOA.

### Diurnal Pattern of Feed-Consumption Behavior

**FIGURE 4:**
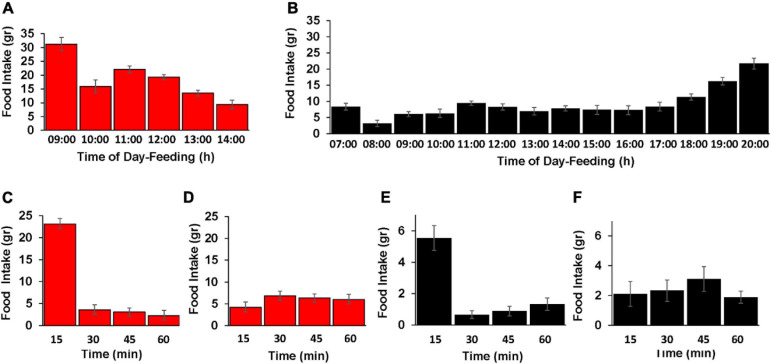
Effect of the TRF on the diurnal pattern of feed-consumption behavior. **(A,B)** Food consumption of the control (CON; black color) and TRF-treated birds (red color) during hour intervals of the free-feeding periods. TRF-treated birds had free access to food between 08:00 and 09:00 h. Control birds consumed food along the lighting time: 06:00–20:00. Each hour period is indicated by the end time (e.g., 08:00–09:00 is indicated by 09:00). **(C–F)** Food intake measured in 15-min intervals for the first and third hours (08:00–09:00 and 11:00–12:00 h, respectively) of eating for the TRF-treated birds (red color) and for the first and fifth hours of eating for the control group (black color, 06:00–07:00 and 11:00–12:00–h, respectively). Vertical lines represent scanning electron microscope. *N* = 28 in each group.

To better understand the feeding behavior in the TRF and control birds, we followed the diurnal pattern of feed consumption along each group’s feeding hours (Figures 4A,B). The general observation was that hourly food intake was higher in the TRF group than in the control group, indicating that signals of lower body energy in the former affected appetite. In both the TRF and control groups, food intake during the first hour of feeding exceeded that of the second hour by 2- and 2.6-fold, respectively, suggesting signals from the digestive system. The observed enhanced feed consumption before dark by the control group is a known phenomenon interpreted as anticipatory behavior ensuring enough feed supply before lights go off (Clark et al., 2019). Our finding that the TRF-treated birds did not increase food intake before their end of food availability suggests that this behavior may be triggered by sensing the reduced day light.

Inspection of eating rate in 15-min intervals (Figures 4C–F) showed that much of the food consumption in the first hour actually occurred within the first 15 min after overnight fasting as reported by McConn et al. (2019) in quails. As a control, we also measured eating in 15-min intervals few hours after the overnight fasting, showing a rather constant rate of food intake in each group. This behavior of high food intake for the first 15 min after fasting suggests signaling from the digestive system in regulating feed intake. Nevertheless, the amount of food intake in the first 15 min likely reflected signaling from other tissues indicating the body’s energy.

## Discussion

Our pioneering study of long-term effects of TRF treatment in commercial laying chickens showed a fundamental difference between the chickens’ response and that of mammals. While mammals immediately (within days) learn to consume amounts of food in the limited time window that are similar to the amounts consumed during *ad libitum* feeding (Currenti et al., 2020), the chickens did not. Food intake was the lowest in the first 9 weeks of treatment and then increased to an average of ∼90% the amount consumed by the *ad libitum*-fed birds, displaying a slow and limited adjustability to the 6 h free-feeding time compared with mammals. Nevertheless, the observed effects of the TRF regimen on egg quality and the recovery of persistent egg production at older age indicated that the TRF treatment delayed some of the negative impacts associated with advanced age.

The most pronounced effect of the reduced energy intake by the TRF chickens was on BW, egg weight, and fat accumulation (reduction of 9.6%, 6.5%, and 2.6-fold, respectively) and to a lesser extent on persistency of egg laying with an average decrease of 2.1 (*P* = 0.005) and 3.8% (*P* = 0.002) egg lay, calculated per hen-housed bird-day, respectively. The lower attenuating effect on egg production compared with the more prominent effects on BW, visceral fat accumulation, and egg weight seems to reflect the selective breeding toward improved rate of egg production for commercial needs. However, this could also reflect a more fundamental natural selective force, compromising BW, fat accumulation, and egg weight but preserving laying efficiency at a time of low food variability. Anyway, persistence of egg lay that was significantly (*P* < 0.05) lowered by the TRF regimen seemed to recover in comparison with the control group, at advanced age (67–86 WOA).

The tendency of delayed deterioration of egg production toward older age in the TRF compared with the control group was accompanied by a delayed decline of egg quality (*P* < 0.05). One of the classical signs of egg quality, the HU score, showed better egg quality at older age (86 WOA) in the TRF group than in the control group. Although the HU score is normalized to egg weight, one may argue that the observed difference in HU scores between the groups relates to the lower average size of the eggs laid by the TRF-treated hens. However, our demonstration that a similar difference in HU score was obtained also by comparing selected eggs of similar size indicated the reliability of this test to egg quality independently of egg size. HU score is known to gradually decrease with layer age by an estimation of 0.38 units per week from the age of 60 weeks onward (Molnar et al., 2016). Eggshell mineralization is also known to deteriorate with aging of layer chickens, resulting in a higher rate of cracked and shell-less eggs (Park and Sohn, 2018). In our experiment, the TRF treatment lowered the proportion of low-quality eggs at older age.

Our follow-up of the diurnal rate of food consumption demonstrated higher hourly food consumption in the TRF group, but not to a level that restored the daily food intake of *ad libitum* consumption. The simplest explanation of this behavior is that the higher rate of feed intake in the TRF group is evoked by signals from the brain and peripheral tissue indicating low body energy. However, the lack of full adjustment of food intake of the control group may result from fullness signals from digestive tissues. These types of signaling were well characterized in mammals, but in mammals, the adipostat activity of leptin has a dominant effect (Friedman and Halaas, 1998). In birds, leptin does not operate as adipostat (Friedman-Einat and Seroussi, 2019), and it seems that birds’ adipose tissue lacks the dominant effect of energy balance control (Bornelov et al., 2018). Nevertheless, other peripheral signaling of energy availability seems to operate in birds since infusion of glucose and lipids to peripheral tissues imposes satiety [for review see Denbow (1994)]. Evidence for appetite control by signaling from the digestive system has also been demonstrated in birds. For example, Dunn et al. (2013) identified a region on chromosome 4 downstream of the cholecystokinin receptor gene, the hypothalamic receptor of the gut hormone cholecystokinin, which controls body growth in broiler-type chickens.

To our knowledge, this is the first study of long-term TRF in layer chickens. Short-term studies of TRF have been reported in geese (Ho et al., 2014; Lui et al., 2014), showing enhancement of feed efficiency by the TRF treatment due to decreasing activity and energy expenditure, as well as a beneficial effect of a two-step TRF (2 h in the morning and 2 h in the evening) with respect to liver expansion (Lui et al., 2014). In another study in layer chickens, the effect of an 8-h TRF regimen for 4 weeks on bird welfare was assessed (Preston, 1987). In that study, a follow-up of cage and agonistic pecking, bouts of feather pecking, and excessive drinking showed some negative and some positive effects, but suggested no serious deterioration of welfare.

Improvement of egg quality of laying hens in old age is considered a bottleneck in achieving “long-lived” layers (Bain et al., 2016). Our study suggests a new approach to achieving this goal. On a practical note, the profile obtained for the dynamic rate of food intake suggests that maintaining TRF under similar caloric intake can be achieved by extending the feeding time from 6 to 8 h. Since feed intake in the sixth hour of TRF eating was about 8% of the daily food intake, it is likely that adding 2 h will allow for consumption of the complete amount of food consumed by the *ad libitum* group (an addition of about 10% daily food intake). We expect that this will correct the negative effect of the TRF treatment on the rate of egg laying and allow extending efficient egg production to an older age, thereby improving efficient utilization of resources, reduction in waste, and an overall reduced carbon footprint (Bain et al., 2016).

In summary, this study contributes to our understanding of appetite control in chickens. The recent finding that leptin does not have adipostat activity in birds (Friedman-Einat and Seroussi, 2019) fits well with the observation in the current study showing that 2.6-fold lower fat accumulation in the visceral adipose tissue did not impose higher food intake in the 6-h window of *ad libitum* feeding. Moreover, this study provides the first evidence that TRF regimen in chickens may postpone age-related decline in egg production. Further optimization of the treatment will be needed to estimate the benefit of the treatment to egg production. Nevertheless, this study demonstrates that laying chickens provide an important model system for the study of long-term TRF treatment. We expect that a longer time window of the TRF treatment, such as 8 h instead of 6, will allow close to *ad libitum* feed intake. It is possible that under these conditions, the beneficial effect of the TRF on delayed physiological aging will be better manifested and contribute to agricultural production, understanding the physiology of aging in birds, and provide evolutionary perspective to the studies of TRF and energy balance control.

## Data Availability Statement

The original contributions presented in the study are included in the article/Supplementary material, further inquiries can be directed to the corresponding author.

## Ethics Statement

All experiments and procedures were performed in accordance with the guide for the care and use of laboratory animals published by the United States National Institutes of Health and were approved by the ethics committee on animal use of the Agricultural Research Organization, Volcani Center (protocol no. 127/18).

## Author Contributions

GS collected and analyzed the data and helped in conducting the experiment. DiS and MR conducted the experiments. SY, SD, and HA helped with the design, collection of data, and analysis of data. OF helped to design the experiments. MF-E designed the experiments and the data analysis and wrote the manuscript. All authors contributed to improve the first draft of the manuscript.

## Conflict of Interest

The authors declare that the research was conducted in the absence of any commercial or financial relationships that could be construed as a potential conflict of interest.

## Footnotes

1 https://ibertec.es/docs/articulos/manejo/LTZ-Management-Guide-Alternative-Systems-EN.pdf
